# Impact of acute versus repetitive moderate intensity endurance exercise on kidney injury markers

**DOI:** 10.14814/phy2.13544

**Published:** 2017-12-21

**Authors:** Coen C. W. G. Bongers, Mohammad Alsady, Tom Nijenhuis, Yvonne A. W. Hartman, Thijs M. H. Eijsvogels, Peter M. T. Deen, Maria T. E. Hopman

**Affiliations:** ^1^ Department of Physiology Radboud Institute for Health Sciences Radboud university medical center Nijmegen The Netherlands; ^2^ Department of Physiology Radboud Institute for Molecular Life Sciences Radboud university medical center Nijmegen The Netherlands; ^3^ Department of Nephrology Radboud Institute for Molecular Life Sciences Radboud university medical center Nijmegen The Netherlands; ^4^ Research Institute for Sports and Exercise Sciences Liverpool John Moores University Liverpool United Kingdom

**Keywords:** Kidney Injury Molecule‐1, neutrophil Gelatinase‐Associated Lipocalin, prolonged Exercise, renal Injury

## Abstract

Exercise may lead to kidney injury through several mechanisms. Urinary Kidney Injury Molecule‐1 (uKIM1) and Neutrophil Gelatinase‐Associated Lipocalin (uNGAL) are known biomarkers for acute kidney injury, but their response to repetitive exercise remains unknown. We examined the effects of a single versus repetitive bouts of exercise on markers for kidney injury in a middle‐aged population. Sixty subjects (aged 29–78 years, 50% male) were included and walked 30, 40 or 50 km for three consecutive days. At baseline and after exercise day 1 and 3, a urine sample was collected to determine uNGAL and uKIM1. Furthermore, urinary cystatin C, creatinine, and osmolality were used to correct for dehydration‐related changes in urinary concentration. Baseline uNGAL was 9.2 (5.2–14.7) ng/mL and increased to 20.7 (11.0–37.2) ng/mL and 14.2(8.0–26.3) ng/mL after day 1 and day 3, respectively, (*P* ≤ 0.001). Baseline uKIM1 concentration was 2.6 (1.4–6.0) ng/mL and increased to 5.2 (2.4–9.1) ng/mL (*P* = 0.002) after day 1, whereas uKIM1 was not different from baseline at day 3 (2.9 [1.4–6.4] ng/mL (*P* = 0.52)). Furthermore, both uNGAL and uKIM1 levels were higher after day 1 compared to day 3 (*P* < 0.01). When corrected for urinary cystatin C, creatinine, and osmolality, uNGAL demonstrated a similar response compared to the uncorrected data, whereas differences in uKIM1 between baseline, day 1 and day 3 (*P*_time _= 0.63) were no longer observed for cystatin C and creatinine corrected data. A single bout of prolonged exercise significantly increased uNGAL concentration, whereas no changes in uKIM1 were found. Repetitive bouts of exercise show that there is no cumulative effect of kidney injury markers.

## Introduction

Exercise is a major challenge to whole‐body homeostasis provoking widespread perturbations in cells, tissues, and organs that are caused by or are a response to the increased metabolic activity of contracting skeletal muscles (Hawley et al. 2014). Maintaining an adequate fluid and electrolyte homeostasis is a key role of the kidneys, which is affected by endurance exercise due to exercise‐induced dehydration (Cheuvront et al. 2010). The exercise‐induced redistribution of blood to active body parts (McAllister 1998), causes a decreased renal perfusion, an increased glomerular permeability, and a decreased filtration ratio (Poortmans et al. 1996; Neumayr et al. 2003; Puggina et al. 2014; de Santana et al. 2017). These and other alterations may lead to acute, but transient, kidney injury. Moreover, animal studies demonstrated an increased number of apoptotic cells in distal tubules after cessation of exercise, which is indicative for kidney injury (Podhorska‐Okolow et al. 2004).

Traditional markers for assessing renal function are the estimated glomerular filtration rate (eGFR) and serum creatinine concentration (Bonventre 2008; National Kidney F KDIGO 2012, 2013). These markers are late stage markers for a decreased kidney function, primarily defined as glomerular filtration rate (GFR), and less well‐suited for the detection of initial kidney injury (Slocum et al. 2012). Over the past years, a number of new biomarkers have been identified to detect acute kidney injury in an early stage, of which Kidney Injury Molecule‐1 (KIM1) and Neutrophil gelatinase‐associated lipocalin (NGAL) are best known (Mishra et al. 2005; Bonventre 2008; Devarajan 2008). Previous studies suggested that a single bout of short‐term high intensity exercise (Junglee et al. 2012) or completing a (ultra)marathon (McCullough et al. 2011; Lippi et al. 2012; Mansour et al. 2017) increases urinary excretion of KIM1 (uKIM1) and NGAL (uNGAL). It has been suggested that exercise intensity is the most important factor that impacts on exercise‐induced kidney stress. Literature found a reduced GFR at exercise intensities of 60% and 80%, whereas no difference in pre and postexercise GFR was found at lower exercise intensities (Freund et al. 1991). Therefore, the question raises whether uKIM1 and uNGAL increase after prolonged walking exercise (~70% intensity) as well, which is a more accessible type of exercise for the whole population. In addition, the interpretation of the findings of previous studies is difficult as uKIM1 and uNGAL were only corrected for urinary creatinine concentration, which might be influenced by exercise and in particular exercise‐induced muscle breakdown (Junglee et al. 2012). Therefore, it is hard to distinguish whether observed changes are the effect of exercise or due to an increased urine concentration because of dehydration. In addition, it is unknown whether the kidney's response to repetitive exercise is similar to that of a single bout of prolonged exercise. Since training programs, especially for endurance athletes, consists of exercise bouts on consecutive days, it is relevant to know whether renal function might be affected in a cumulative way.

Therefore, the aim of this study was to examine the effect of a single versus repetitive bouts of prolonged walking exercise in healthy adults on markers for kidney injury in order to establish the physiological responses of the kidneys to prolonged and repeated endurance exercise. To account for exercise‐induced changes in urinary concentration, uNGAL and uKIM1 will be corrected for cystatin C, creatinine, and osmolality. We hypothesized that a single bout of endurance exercise causes an increase in biomarkers for kidney injury, whereas a cumulative kidney injury biomarker response will be observed after three consecutive days of prolonged exercise.

## Methods

### Subjects

A total of 60 subjects (aged 29–78 years, 30 men and 30 women) participated in this prospective intervention study. All subjects participated in the largest walking march in the world (i.e., the International Nijmegen Marches), in which subjects walked at a self‐selected pace for 30 km (*n* = 13*),* 40 km (*n* = 37) or 50 km (*n* = 10) on consecutive days. Subjects with self‐reported kidney disease were excluded from study participation. The study was approved by the Medical Ethical Committee of the Radboud University Medical Center, and all subjects gave written informed consent prior to participation in the study.

### Experimental design

Data were collected at baseline (12–36 h before the start of the walking event) and directly after walking day 1 and 3. Baseline measurements were conducted in controlled laboratory conditions and consisted of assessment of subject characteristics (i.e., age, body mass index, fat percentage, blood pressure, and habitual physical activity levels) and collection of a blood and urine sample for the assessment of fluid balance, eGFR and kidney injury parameters. Prior to the onset of exercise at day 1 and 3, body mass was assessed. Subsequently, subjects were instructed to walk at a self‐selected pace. Heart rate was monitored every 5 km during the first walking day, and subjects were asked to register their fluid intake during the whole exercise bout. Directly after the finish of day 1 and 3, body mass was measured and a blood and urine sample was taken.

### Measurements

#### Subject characteristics

At baseline, body mass (SECA 888 scale, Hamburg, Germany) and body height were measured, which were used to calculate the body mass index (BMI). Furthermore, body fat percentage was calculated using a four‐point (biceps, triceps, sub‐scapular, and sub‐iliac) skinfold thickness measurement (Durnin and Womersley 1974). Thereafter, blood pressure and heart rate were measured twice using an automated sphygmomanometer (M5‐1 intellisense, Omron Healthcare, Hoofddorp, the Netherlands) after 5 min of supine rest. Finally, all subjects completed a questionnaire regarding their habitual physical activity levels, including the hours of exercise per week, and the walking specific training history in the year prior to the walking march.

#### Blood sample

A venous blood sample was taken to determine plasma levels of sodium, hematocrit, and hemoglobin (Rapidpoint^®^ 400, Siemens Health Care Diagnostics Inc., Tarrytown, New York) at baseline and directly after exercise cessation. Using changes in plasma hematocrit and hemoglobin concentration the relative plasma volume change (in %) was calculated according to the formula by Dill and Costill (1974). Hypo‐ and hypernatremia were defined as plasma sodium concentrations of ≤135 and ≥145 mmol/L, respectively (Adrogue and Madias 2000; Hew‐Butler et al. 2005). Furthermore, serum creatinine was measured at baseline and directly after completion of the exercise on day 1 and day 3. The estimated glomerular filtration ratio (eGFR) was calculated to assess baseline kidney function using the CKD‐EPI creatinine equation, which contained the serum creatinine concentration as well as subject's age, gender, and ethnicity (Levey et al. 2009). Furthermore, plasma interleukin‐6 (IL‐6) and tumor necrosis factor‐*α* (TNF‐*α*) were measured to examine differences in inflammatory response between baseline and postexercise.

#### Urine sample

At baseline and directly after exercise cessation, all subjects provided a urine sample to examine kidney injury and fluid balance. Urinary cystatin C concentration was measured using the nephelometric method (Behring Nephelometer II, Siemens Healthcare, Den Haag, The Netherlands). Furthermore, the urinary creatinine concentration was measured using the enzymatic method (Cobas C6000, Roche Diagnostics, Indianapolis). Urine osmolality was examined using an osmometer (Advanced Model 3320 Micro‐Osmometer; Osmometer, Advanced Instruments, Norwood). Urine specific gravity and urine proteinuria was assessed using a dip‐stick method (Clinitek Status^®^ Analyzer, Siemens Healthcare Diagnostics Inc., Tarrytown, New York). Proteinuria was defined, using a categorical scale: negative (0 g/L), trace (0.15 g/L), + (0.3 g/L) or ++(1 g/L).

In order to determine kidney injury in response to exercise, we measured urinary concentrations of KIM1 and NGAL (both monomeric and dimeric) in duplicate using the previously described sandwich ELISA assay (E‐EL‐H0186 and E‐EL‐H0096, Elabscience Biotechnology, Wuhan, China) (Han et al. 2002; van Timmeren et al. 2007). Furthermore, uKIM1 and uNGAL concentrations were corrected for urinary cystatin C, creatinine, and osmolality. The corrected uKIM1 and uNGAL data were calculated by dividing the individual uncorrected data by the corresponding cystatine C and creatinine levels and the urine osmolality. Furthermore, acute kidney injury was defined based on the Acute Kidney Injury Network (AKIN) criteria (Mehta et al. 2007). Stage 1 was defined as a 1.5–2‐fold or 26.4 *μ*mol/L increase in serum creatinine concentration from baseline to peak value on either day 1 or day 3, whereas stage 2 was defined as a 2–3‐fold increase in serum creatinine concentration (Mehta et al. 2007).

#### Fluid balance

All subjects received written and individual oral instructions about the registration of their fluid intake in a diary. Subjects were allowed to drink ad libitum, as long as they registered the time (in blocks of 1 h), amount (standardized sized cups, bottles, etc.) and type (water, sports drink, other) of their individual fluid intake 12 h prior to the start and throughout the walking exercise. Furthermore, body mass was measured prior to and directly after finishing the walking march. Relative change in body mass (in %) between both measurements was calculated. We defined dehydration as a relative body mass loss ≥2% (Goulet 2013).

#### Exercise intensity

Heart rate was measured at every 5 km milestone during the first walking day using a 2‐channel chest band (Polar Electro, Oy, Kempele, Finland), which was instrumented to the subjects prior to the start. The average of at least three consecutive heart rate measurements (~15 sec) was taken at every 5 km point while walking. Measurements prior to the start and directly after finishing were excluded for the calculation of the mean heart rate during exercise. Exercise intensity (%) was calculated by dividing the mean heart rate by the predicted maximal heart rate according to Tanaka's formula (HR_max_ = 208–0.7*age) (Tanaka et al. 2001).

#### Ambient conditions

The dry bulb, wet bulb, and globe temperature, as well as the relative humidity were measured every 30 min throughout the experiment using a portable climate monitoring device (Davis instruments inc., Hayward), which was located at the start/finish area. Based on the above mentioned temperatures, the wet bulb globe temperature (WBGT) was calculated using the formula: WBGT = 0.1(T_dry bulb_) + 0.7(T_wet bulb_) + 0.2(T_globe_) (Armstrong et al. 2007).

### Statistical analysis

Normally distributed data were presented as mean ± SD, whilst non‐Gaussian distributed data were presented as median (interquartile range). The statistical analysis was conducted using the Statistical Package for Social Sciences (SPSS version 20, Armonk, NY), in which the level of significance was set at *P* < 0.05. Data were checked for normality using the Shapiro–Wilk test. In case of non‐Gaussian distributed data, the statistical analysis was performed using the nonparametric equivalents. The effect of acute exercise on kidney injury and fluid balance was examined using a paired Students’ *T*‐test or a Wilcoxon signed‐rank test. A repeated measures ANOVA was used to assess differences in kidney injury and fluid balance over time, which was used to determine the effects of repetitive exercise. Subsequently, a post hoc Bonferroni test was used to determine differences between individual experimental days. In case of Non‐Gaussian distributed data a Friedman test was used to assess differences in kidney injury over time, followed by a Wilcoxon signed rank test to determine differences between individual days. A Kruskal–Wallis test was used to determine sex differences in kidney response to acute and repetitive bouts of exercise. A Chi‐square test was used to determine differences in the prevalence of dehydration, hypo‐ or hypernatremia, high urine specific gravities and proteinuria.

## Results

### Subject and Exercise characteristics

At baseline the average eGFR was 89.3 ± 11.6 mL/(min·1.73 m^2^) (Table 1). All subjects successfully completed the walking exercise bouts. An overview of baseline characteristics is shown in Table 1. Exercise duration and walking speed did not differ between both experimental days (Table 2). Moreover, subjects completed the exercise bout at 71 ± 9% of their predicted maximal heart rate, and walked for 8–9 h with an average walking speed of 4.8 km/h. WBGT ranged between 13°C (04.00 am) and 24°C (17.00 pm), and average WBGT was higher on day 1 versus 3 (21.1°C vs. 17.6°C, *P* < 0.001), whereas the relative humidity did not differ between both days (*P* = 0.08). Elevated plasma IL‐6 and TNF‐*α* levels were found after both exercise day 1 and day 3 compared to baseline (all *P* < 0.05), whereas IL‐6 and TNF‐*α* were higher after day 1 compared to day 3 (*P* < 0.001, Table 2). In addition, no correlation was found between inflammatory markers and corrected as well as uncorrected uNGAL concentrations (all *P* > 0.05) after day 1 and day 3.

**Table 1:** Baseline subject characteristics (*n* = 60)

Parameter	Value
Sex (male:female)	30: 30
Age (years)	56 ± 10
Length (m)	173 ± 8
Body mass (kg)	75.4 ± 13.8
BMI (kg/m^2^)	24.9 ± 3.0
Fat percentage (%)	31.2 ± 5.5
Systolic blood pressure (mmHg)	138 ± 20
Diastolic blood pressure (mmHg)	85 ± 11
Resting heart rate (bpm)	63 ± 7
eGFR (mL/min·1.73 m^2^)	89.3 ± 11.6
Activity score (au)	6965 ± 3462
MET min a day (MET min)	1027 ± 502

Subject characteristics for the total group. Data were presented as mean ± SD. MET, Metabolic equivalent of task; bpm, beats per minute; au, arbitrary unit.

**Table 2:** Exercise characteristics and fluid balance parameters at baseline and after day 1 and day 3

	Baseline	Day 1	Day 3	*P*‐value
Exercise characteristics				
Exercise duration (hh:mm)	–	8:10 ± 1:59	8:28 ± 1:53	0.29
Average HR (bpm)	–	112 ± 14	–	–
Exercise intensity (% of HR max)	–	70.8 ± 8.9	–	–
Walking speed (km/h)	–	4.8 ± 0.7	4.7 ± 0.7	0.22
WBGT (°C)	–	21.1 ± 1.5	17.6 ± 2.5^b^	**<0.001**
Relative humidity (%)	–	71.4 ± 14.9	67.5 ± 19.6	0.08
Fluid balance				
Fluid intake (mL/h)	–	309 ± 121	300 ± 133	0.48
Water (%)	–	50.7 ± 22.2	48.1 ± 21.0	0.26
Other (%)	–	49.3 ± 22.2	51.9 ± 21.0	0.26
Urine osmolality (mOsm/mL)^c^	890 (589–1113)	998 (719–1159)	945 (649–1264)	0.36
Hemoglobin (mmol/L)	15.2 ± 1.4	15.5 ± 1.5^a^	14.8 ± 1.5^a^, ^b^	**<0.001**
Hematocrit (L/L)	44.8 ± 4.2	45.6 ± 4.4^a^	43.5 ± 4.5^a^, ^b^	**<0.001**
Plasma volume change (%)	–	–2.9 ± 8.5	5.8 ± 10.7^b^	**<0.001**
Plasma sodium (mmol/L)	141.2 ± 1.6	143.4 ± 2.4^a^	141.4 ± 2.0^b^	**<0.001**
Prevalence of hyponatremia (n(%))	0 (0%)	0 (0%)	0 (0%)	1.00
Prevalence of hypernatremia (n(%))	1 (1.7%)	18 (30%)^a^	2 (3.3%)^b^	**<0.001**
Body mass change (kg)	–	−0.8 ± 1.0	−0.4 ± 0.5^b^	**0.001**
Body mass change (%)	–	−0.9 ± 1.2	–0.4 ± 0.7^b^	**0.005**
Dehydration; ≥2% body mass loss (n(%))	–	12 (20%)	0 (0%)^b^	**<0.001**
Inflammation				
Plasma IL‐6 (pg/mL)	0.5 (0.3–0.6)	5.2 (3.4–8.4)^a^	2.0 (1.5–3.5)^a^, ^b^	**<0.001**
TNF‐*α* (pg/mL)	1.53 (1.3–1.8)	1.6 (1.4–1.9)^a^	1.5 (1.3–1.7)^a^, ^b^	**<0.001**

Exercise characteristics and fluid balance at baseline, after exercise day 1 and day 3. Data were presented as mean ± SD or median (IQR). *P*‐values represents the results of the one‐way ANOVA or nonparametric alternative.

a Significantly different from baseline.

b Significantly different from day 1.

c A nonparametric alternative was used: Friedman test for time‐effect and Wilcoxon signed‐ranks test for differences between individual days.

### Fluid balance

Fluid balance data are shown in Table 2. A significant decrease in body mass was found after both, day 1 and day 3 (both *P* < 0.001). Body mass losses were significantly larger at day 1 (−0.9 ± 1.2%) compared to day 3 (−0.4 ± 0.7%, *P* = 0.005). In line with this, 12 subjects (20%) were considered dehydrated after day 1 (body mass loss ≥2%), while none of the subjects were dehydrated after day 3 (*P* < 0.001). Furthermore, we demonstrated an exercise‐induced increase in urine specific gravity on both, day 1 and day 3 (*P* = 0.005 and *P* = 0.025 respectively), with a comparable incidence of urine specific gravity levels ≥1.020 g/mL on day 1 compared to day 3 (*P* = 0.54). Postexercise plasma hemoglobin and hematocrit levels were increased compared to baseline (both *P* < 0.001), in which hemoglobin and hematocrit levels were higher after day 1 compared to day 3 (both *P* < 0.001). Furthermore, plasma sodium concentration and the prevalence of hypernatremia were increased after day 1, but not after day 3 (both *P* < 0.001).

### Kidney injury – uncorrected data

**Figure 1:**
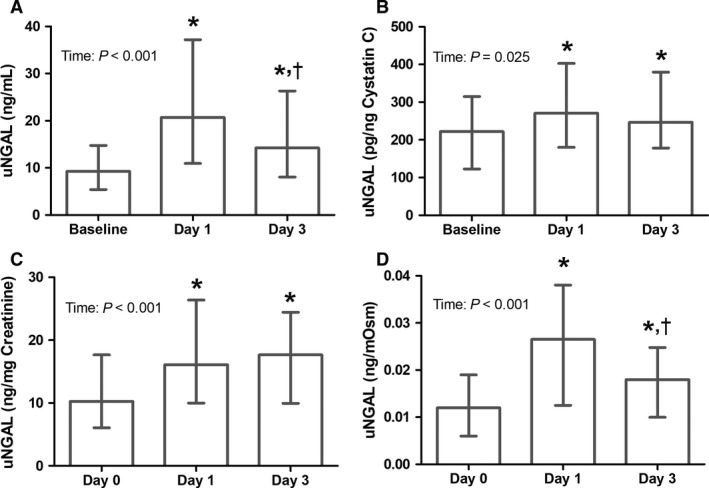
Effect of acute and repetitive bouts of prolonged exercise on uNGAL concentration (*n* = 60). The uncorrected data are presented (A), as well as after correction for cystatin C (B), creatinine (C) and osmolality (D). A significant increase in NGAL concentration compared to baseline was found after day 1 and 3. A Friedman test was used to examine differences over time, whereas a Wilcoxon signed‐rank test was used to assess differences between days. Data were presented as median with interquartile range. *Represents a significant difference (*P* < 0.05) from baseline.

**Figure 2:**
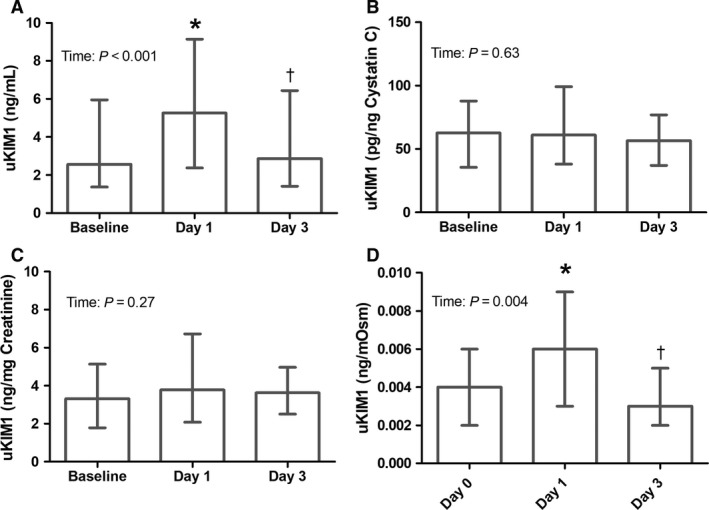
Effect of acute and repetitive bouts of prolonged exercise on uKIM1 concentration (*n* = 60). The uncorrected data are presented (A), as well as after correction for cystatin C (B), creatinine (C) and osmolality (D). Increased uKIM1 levels after day 1 compared to baseline were found for the uncorrected and osmolality corrected data, whereas no differences in uKIM1 between days were found after cystatin C and creatinine correction. A Friedman test was used to examine differences over time, and a Wilcoxon signed‐rank test was used to assess differences between days. Data were presented as median with interquartile range. * Represents a significant difference (*P* < 0.05) from baseline, and † represents a difference from day 1.

Absolute median uNGAL concentration was 9.2 (5.2–14.7) ng/mL at baseline and increased postexercise to 20.7 (11.0–37.2) ng/mL and 14.2 (8.0–26.3) ng/mL on day 1 and day 3, respectively, (both *P* ≤ 0.001, Fig. 1A). Uncorrected uNGAL levels after day 1 were significantly higher compared to day 3 (*P* < 0.001). For uncorrected uKIM1, median baseline concentration was 2.6 (1.4–6.0) ng/mL and increased to 5.2 (2.4–9.1) ng/mL (*P* = 0.002, Fig. 2A) after exercise day 1 and did not differ from baseline after day 3 (2.9 (1.4–6.4) ng/mL (*P* = 0.52)). Furthermore, uncorrected uKIM1 concentration after day 1 was higher compared to day 3 (*P* = 0.003).

### Kidney injury – cystatin C corrected data

Urinary cystatin C concentration was significantly elevated after day 1 (0.09 (0.05–0.12 mg/L)) compared to baseline (0.05 (0.03–0.08 mg/L); *P* < 0.001), but this effect disappeared at day 3 (0.06 (0.03–0.09 mg/L); *P* = 0.28). Cystatin C corrected uNGAL concentration at baseline was 222 (134–316) pg/ng cystatin C, and increased to 271 (180–403) pg/ng cystatin C (*P* = 0.002) and 247 (178–379) pg/ng cystatin C (*P* = 0.010) after day 1 and day 3, respectively, (*P*_time _= 0.025, Fig. 1B). Postexercise uNGAL concentration did not differ between day 1 and day 3 (*P* = 0.75). A total of 39 (65%) subjects demonstrated elevated uNGAL levels after day 1 and/or day 3, but no differences between day 1 and day 3 were observed (*P* = 1.00). uKIM1 corrected for urinary cystatin C concentration showed no differences across days (*P*_time _= 0.63, Fig. 2B).

### Kidney injury – creatinine corrected data

Urinary creatinine concentration was significantly elevated after day 1 (1.31 (0.89–2.02 mg/mL)) compared to baseline (0.92 (0.61–1.50 mg/mL); *P* < 0.001), but this effect disappeared at day 3 (0.77 (0.52–1.48 mg/mL); *P* = 0.80). Creatinine corrected uNGAL concentration at baseline was 10.3 (6.1–17.7) ng/mg creatinine, and increased to 16.1 (10.0–26.4) ng/mg creatinine (*P* < 0.001) and 17.7 (9.9–24.4) ng/mg creatinine (*P* < 0.001) after day 1 and day 3, respectively, (*P*_time _< 0.001, Fig. 1C). Postexercise creatinine corrected uNGAL concentration did not differ between day 1 and day 3 (*P* = 0.48). uKIM1 corrected for urinary creatinine concentration showed no differences across days (*P*_time _= 0.27, Fig. 2C).

### Kidney injury – osmolality corrected data

Baseline urine osmolality was 890 (589–1113 mOsm/mL) and demonstrated a nonsignificant (*P* = 0.36) increase after completion of the exercise bout on both day 1 (998 (719–1159 mOsm/mL)) and day 3 (945 (649–1264 mOsm/mL)). Baseline osmolality corrected uNGAL concentration was 0.012 ng/mOsm, with increased levels after day 1 (0.027 (0.013–0.038) ng/mOsm) and after day 3 (0.18 (0.010–0.025) ng/mOsm) (*P*_time _< 0.001, Fig. 1D). Furthermore, osmolality corrected uNGAL levels after day 1 were significantly higher compared to day 3 (*P* < 0.001). Osmolality corrected uKIM1 was 0.004 (0.002–0.006 ng/mOsm) at baseline and increased to 0.006 (0.003–0.009 ng/mOsm) after exercise day 1 (*P* = 0.003) and did not differ from baseline after day 3 (0.003 (0.002–0.005) ng/mOsm, *P* = 0.49, Fig. 2D). Furthermore, osmolality corrected uKIM1 concentration after day 1 was higher compared to day 3 (*P* = 0.002).

### Kidney injury – AKIN criteria and eGFR

Serum creatinine concentration was significantly elevated after day 1 (86.9 ± 22.8 *μ*mol/L) compared to baseline (75.3 ± 13.7 *μ*mol/L; *P* < 0.001), but this effect disappeared at day 3 (75.6 ± 14.9 *μ*mol/L; *P* = 0.81). Moreover, 10% of the subjects (*n* = 6) developed stage 1 acute kidney injury by AKIN criteria after day 1, whereas none of the subjects developed stage 1 acute kidney injury after day 3. In addition, none of the subjects developed stage 2 kidney injury. At baseline the average eGFR was 89.3 ± 11.6 mL/(min·1.73 m^2^)which decreased after day 1 to 79.4 ± 16.3 mL/(min·1.73 m^2^)(*P* < 0.001), but no difference was found after day 3 (89.8 ± 13.9 mL/(min·1.73 m^2^); *P* = 0.70). Furthermore, *n* = 2 subjects were traced with proteinuria at baseline (Table 3), *n* = 8 subjects after day 1 (range: 0.15–1 g/L) and *n* = 5 after day 3 (range: 0.15–0.30 g/L, *P* = 0.39).

**Table 3:** Renal injury markers at baseline and after day 1 and day 3

	Baseline	Day 1	Day 3	*P*‐value
Urine Cystatin C (mg/L)^c^	0.05 (0.03–0.08)	0.09 (0.05–0.12)^a^	0.06 (0.03–0.09)	<0.001
Urine Creatinine (mmol/L)^c^	8.1 (5.4–13.3)	11.6 (7.9–17.9)^a^	6.8 (4.6–13.1)^b^	<0.001
Serum Creatinine (*μ*mol/L)	75.3 ± 13.7	86.9 ± 22.8^a^	75.5 ± 15.0^b^	<0.001
Proteinuria				0.39
Negative (0 g/L) (n(%))	58 (96.7%)	52 (86.7%)	55 (91.7%)	
Trace (0.15 g/L) (n(%))	2 (3.3%)	3 (5%)	2 (3.3%)	
+(0.3 g/L) (n(%))	0 (0%)	4 (6.7%)	3 (5%)	
++(1 g/L) (n(%))	0 (0%)	1 (1.7%)	0 (0%)	

Renal injury markers at baseline, after exercise day 1 and day 3. Data were presented as median (IQR).

a Significantly different from baseline.

b Significantly different from day 1.

c A nonparametric alternative was used: Friedman test for time‐effect and Wilcoxon signed‐rank test for differences between individual days.

### Subject and exercise characteristics associated with kidney injury

The increase in uncorrected and osmolality corrected uNGAL and uKIM1 in response to acute exercise was higher for men compared to women (all *P* < 0.05), while no differences were found for cystatin C and creatinine corrected data. After repetitive bouts of prolonged exercise no differences were found between men and women (all *P* > 0.05), except for a lower creatinine corrected uNGAL concentration for men compared to women (*P* = 0.005). Furthermore, for both men and women no correlation was found between age and uNGAL/uKIM1 levels (uncorrected and corrected) after both acute and prolonged exercise. In addition, the physical activity level of the subjects was not related to kidney injury outcomes. No relationship was found between kidney injury and walking distance or exercise duration after both acute and repetitive bouts of prolonged exercise. Furthermore, the average exercise intensity during a single bout of prolonged exercise was not related to the uKIM1 concentration and creatinine and cystatin C corrected uNGAL, whereas a higher exercise intensity is related to higher uncorrected and urine osmolality corrected uNGAL levels (both *R* ^2 ^= 0.27, *P* = 0.043).

## Discussion

The purpose of this study was to examine the effects of acute as well as repetitive bouts of prolonged exercise on markers of kidney injury. When corrected for exercise‐induced changes in hydration status, we found that an acute bout of prolonged exercise resulted in tubular kidney injury, as evidenced by significant increase in uNGAL. In contrast to our hypothesis, we found no differences in renal responses between an acute bout versus repetitive bouts of exercise on three consecutive days, which indicates that there is no cumulative effect of kidney injury markers in response to repetitive exercise.

We found an increased uNGAL concentration after performance of single and repetitive bouts of exercise, which is indicative for acute kidney injury, and more specifically tubular injury (Mishra et al. 2005; Devarajan 2008). In a normal situation, NGAL is produced continuously at low levels by neutrophils of different tissues (i.e., colon‐, trachea‐, and kidney epithelium), including the distal tubules (Schmidt‐Ott 2011; Martensson and Bellomo 2014). The circulating NGAL is filtered by the glomerulus and reabsorbed by the proximal tubule, resulting in low urine concentrations (Schmidt‐Ott 2011; Helanova et al. 2014). In case of acute kidney injury, the proximal tubular uptake of NGAL is impaired and the NGAL expression and release is upregulated in the distal tubule (Devarajan 2008; Schmidt‐Ott 2011); both will increase the urinary excretion of NGAL (Schmidt‐Ott 2011), while the upregulated secretion of NGAL in the distal tubule is the primary source (Helanova et al. 2014). A cross‐sectional study demonstrated that subjects with acute tubular necrosis had a more than 25‐fold higher uNGAL concentration (normalized for urinary creatinine) compared to matched healthy controls (570 vs. 23 ng/mL, respectively) (Mori et al. 2005). We found a relatively small increase in uncorrected uNGAL (2.3‐fold higher, 9.2 ng/mL at baseline to 20.7 ng/mL at day 1). Moreover, both baseline and postexercise uNGAL concentrations were below the previously determined normal (23 ng/mL) and cut‐off value (104 ng/mL) for acute kidney injury, in which acute kidney injury is defined as a twofold increase in serum creatinine or a 50% decrease in GFR (Bellomo et al. 2004; Nickolas et al. 2012).

In a previous study the effect of marathon running (McCullough et al. 2011) on uncorrected uNGAL concentrations was examined. They found a 5.7‐fold increase in uNGAL concentration postexercise compared to baseline (47.0 ng/mL). Our uncorrected values showed a 2.3 and 1.5‐fold increase after day 1 and day 3, respectively. The lower average exercise intensity (71% vs. 80%) and lower fluid balance disturbances (−0.9% vs. −1.7%) in our study may partly explain the smaller increase in uNGAL (Freund et al. 1991; Neumayr et al. 2003) compared to the observations in marathon runners. After correction for urine cystatin C concentration the uNGAL concentration showed a 1.2‐fold increase, which suggests an even lower level of kidney injury. Therefore, our results suggest that an acute bout of exercise results in minor tubular kidney injury.

In contrast to uNGAL, uKIM1 levels did not differ between baseline and both exercise days after correction for cystatin C and creatinine. KIM1 is a type‐1 transmembrane protein, which is absent in normal conditions, but elevated in the proximal tubule apical membrane cells after injury (van Timmeren et al. 2007; Shao et al. 2014). In a previous study in marathon runners, postexercise uKIM1 concentration was higher compared to baseline (3.5 ± 1.6 ng/mL vs. 2.6 ± 1.6, respectively) (McCullough et al. 2011). Although kidney injury may have occurred, the reported increase may not be realistic as these data was not corrected for changes in hydration status. Our data underline the importance of this correction, as we found an increased uncorrected uKIM1 concentration after exercise day 1 as well, but the increase disappeared after cystatin C and creatinine corrections were applied. Moreover, with a similar correction for hydration status in the marathon runners, the exercise‐induced increase in uKIM1 probably disappears. As in our study we found that creatinine and cystatin C corrected uKIM1 levels were not affected by a single or repeated bouts of moderate intensity endurance exercise, we conclude that exercise does not induce temporary injury to the proximal tubules.

The discrepancy in uNGAL and uKIM1 responses to exercise might be explained by a difference in etiology of release of both markers. KIM1 is undetectable in urine of normal kidneys (Chiusolo et al. 2010) and the expression is increased in damaged proximal tubular cells and tubular inflammation (van Timmeren et al. 2007), whereas increased NGAL levels are associated with reduced proximal tubular uptake and injury of both the proximal and distal tubules (Schmidt‐Ott 2011). The discrepancy between changes in concentrations of uKIM1 and uNGAL may indicate that, due to energetic stress, exercise‐induced kidney injury primarily affects the distal tubule, leading to elevated secretion of NGAL (Helanova et al. 2014). In animal studies it was demonstrated that acute exercise, which consists of running on a treadmill at 1 km/h until exhaustion (mean time to exhaustion = 90 min), resulted in a significant increase in apoptotic cells in the kidney 2, 6 and 96 h after cessation of exercise (Podhorska‐Okolow et al. 2004, 2006). Moreover, the apoptosis was only present in the distal tubulus and collecting duct of all exercising rats, whereas the presence of apoptosis was not demonstrated in proximal tubular cells (Podhorska‐Okolow et al. 2004, 2006). The authors suggested that the increased distal tubular apoptosis might be explained by transient renal ischemia as well as an increased expression of angiotensin II receptor type 1 and type 2 in distal tubular cells (Podhorska‐Okolow et al. 2006). Moreover, after ischemic injury in the kidneys, apoptotic cells were prominent in distal tubulus (Oberbauer et al. 2001). The increased exercise‐induced apoptosis of distal tubular cells, might explain the different response of uKIM1 and uNGAL in our study.

In contrast to our hypothesis, the uNGAL and uKIM1 concentration did not further increase after the first day of prolonged walking exercise. This may possibly be explained by the relatively short plasma half‐life time of both NGAL (~15 min) (Pickering and Endre 2014) and KIM1 (~6 h) (Bailly et al. 2002), which would cause uNGAL and uKIM1 concentrations to drop back to baseline before onset of exercise on consecutive days. Furthermore, recent studies assessing myocardial dysfunction, using serum B‐type Natriuretic Peptide (Aengevaeren et al. 2016) or serum troponin (Eijsvogels et al. 2010) *(*half‐life time ~90–120 min*),* after four consecutive days of prolonged walking exercise did not find a difference in postexercise values across the 4 days. Therefore, no accumulative effect of repetitive exercise was found with respect to myocardial dysfunction, which is in line with our results in which we demonstrate a comparable response of kidney injury to repetitive prolonged walking exercise on consecutive days.

We are the first to apply urinary cystatin C to correct for changes in hydration status. However, urinary cystatin C levels might be slightly elevated by exercise‐induced proximal tubular stress (Conti et al. 2006), which questions the role as correction factor. However, the baseline and postexercise urinary cystatin C levels found in our study are very low and fit well within the normal range (0.03–0.3 mg/L) described for nonexercising healthy subjects (Uchida and Gotoh 2002; Conti et al. 2006). These data underscore our indication above that the proximal tubular function is not affected in our two exercise bouts. Although the urinary creatinine concentration is commonly used in literature to correct for changes in hydration status, its application in exercise settings is less suitable due to the physiological muscle breakdown and related creatinine release during exercise (Junglee et al. 2013). Therefore, under nonsteady‐state conditions, such as exercise, the urinary creatinine concentration changes over time, which probably affects the normalized level of both biomarkers (Waikar et al. 2010). The exercise‐induced increase in urinary creatinine concentration after day 1 might, therefore, be caused by both dehydration and muscle breakdown. Furthermore, as a result of the high baseline and postexercise urine osmolality and the less controlled exercise conditions with different fluid and salt intake along subjects, the urine osmolality is our study design less suitable to normalize for changes in hydration status.

### Clinical implications

A small exercise‐induced increase in both corrected and uncorrected uNGAL was found after both day 1 and day 3, whereas corrected postexercise uKIM1 levels did not differ from baseline. The uncorrected uNGAL levels, however, were below described cutoff values for acute kidney injury (Nickolas et al. 2012). Our data, therefore, suggest that a single bout as well as repetitive bouts of exercise in subjects who are not diagnosed with kidney disease do lead to some kidney injury, but not to acute kidney injury. Furthermore, exercise‐induced inflammation, which is represented by increased plasma IL‐6 and TNF‐*α* levels after exercise on day 1 and day 3, may also contribute to elevated plasma NGAL and consequently uNGAL concentrations (Abella et al. 2015). However, we did not find a correlation between uNGAL concentration and the inflammatory markers IL‐6 and TNF‐*α*, which suggests that the increase in uNGAL is not associated with exercise‐induced changes in inflammatory status. Furthermore, based on current study it is hard to establish whether the changes in uNGAL are the result of renal responses to the stress of exercise or the result of “temporary” kidney injury or a combination of both.

### Limitations

The strength of this study is the inclusion of a large group of subjects that performed prolonged exercise on three consecutive days, in which the urinary outcome parameters were corrected for dehydration‐related changes in urine concentration. However, there are some limitations that should be taken into account. It is previously suggested that urine normalization to urinary flow rates is the best option to correct urinary outcomes for changes in hydration status. However, obtaining the urinary flow rate in field based settings is very hard and inconvenient to the subjects. Second, spot urines were used for all laboratory analyses. Although spot urines are well correlated with 24 h urine samples and have the potential to operate as a surrogate for the preferred 24 h urine collection, the use of spot urines is less accurate compared to a 24 h urine collection (van Huysduynen et al. 2014). However, urine collection during these walking marches is highly inconvenient to the subjects. Third, the average eGFR at baseline was 89.3 ± 11.6 mL/(min·1.73 m^2^). This relatively low eGFR might be explained by the previously described decrease in eGFR with aging (National Kidney F, 2002).

In conclusion, an acute bout of prolonged moderate intensity exercise does not impact on uKIM1 levels corrected for hydration status in a middle‐aged population, but does increase uNGAL, suggesting distal tubular injury. Moreover, no differences in renal responses are present between an acute bout versus repetitive bouts of exercise on three consecutive days, which indicates that there is no cumulative effect of kidney injury markers in response to repetitive exercise.

## Conflicts of Interest

None declared.
